# Real-world experience with cefiderocol therapy for *Pseudomonas aeruginosa* and other multidrug resistant gram-negative infections within the Veterans Health Administration, 2019–2022

**DOI:** 10.1017/ash.2023.165

**Published:** 2023-05-04

**Authors:** Andrew Chou, David Ramsey, Eva Amenta, Barbara W. Trautner

**Affiliations:** 1 Michael E. DeBakey, Veterans’ Affairs Medical Center, Center for Innovations in Quality, Effectiveness, and Safety (IQuESt), Houston, Texas; 2 Section of Infectious Diseases, Department of Medicine, Baylor College of Medicine, Houston, Texas; 3 Section of Health Services Research, Department of Medicine, Baylor College of Medicine, Houston, Texas

## Abstract

**Objective:**

Single-center and regional studies have reported outcomes after treatment with cefiderocol, a novel siderophore cephalosporin. We report on real-world use, clinical outcomes, and microbiological outcomes with cefiderocol therapy within the Veterans’ Health Administration (VHA).

**Design:**

Prospective, observational descriptive study.

**Setting:**

Veterans’ Health Administration, 132 sites across the United States, during 2019–2022.

**Patients:**

This study included patients admitted to any VHA medical center who received cefiderocol for ≥2 days.

**Methods:**

Data were obtained from the VHA Corporate Data Warehouse and through manual chart review. We extracted clinical and microbiologic characteristics and outcomes.

**Results:**

In total, 8,763,652 patients received 1,142,940,842 prescriptions during the study period. Of these, 48 unique individuals received cefiderocol. The median age of this cohort was 70.5 years (IQR, 60.5–74), and the median Charlson comorbidity score was 6 (IQR, 3–9). The most common infectious syndromes were lower respiratory tract infection in 23 patients (47.9%) and urinary tract infection in 14 patients (29.2%). The most common pathogen cultured was *P. aeruginosa* in 30 patients (62.5%). The clinical failure rate was 35.4% (17 of 48), and 15 (88.2%) of these 17 patients died within 3 days of clinical failure. The 30-day and 90-day all-cause mortality rates were 27.1% (13 of 48) and 45.8% (22 of 48), respectively. The 30-day and 90-day microbiologic failure rates were 29.2% (14 of 48) and 41.7% (20 of 48), respectively.

**Conclusions:**

In this nationwide VHA cohort clinical and microbiologic failure occurred in >30% of patients treated with cefiderocol, and >40% of these died within 90 days. Cefiderocol is not widely used, and many of the patients who received it had substantial comorbidities.

Cefiderocol is a novel siderophore cephalosporin for the treatment of gram-negative infections, including isolates that are resistant to carbapenems and that produce metallo-β-lactamases.^1,2^ The CREDIBLE-CR phase 3 randomized clinical trial comparing treatments of carbapenem-resistant gram-negative infections found that treatment with cefiderocol was associated with similar clinical and microbiological outcomes in comparison to treatment with best available therapy.^2^ However, the cefiderocol group had a higher mortality rate than the best-available-therapy group, primarily in the subset of patients with *Acinetobacter* spp infection. Subsequently published studies did not find cefiderocol therapy to be associated with increased mortality in nosocomial pneumonia or urinary tract infections.^3–5^ Since its approval, real-world use of cefiderocol has mainly been reported for therapy of infections by *Acinetobacter baumannii* but less is known about outcomes for treatment of other gram-negative infections.^6–16^ One published study of 17 patients reported outcomes of treating *Pseudomonas aeruginosa* infections with cefiderocol, but this study was conducted under compassionate use criteria,^12^ which do not fully reflect the uses and outcomes of on- and off-label use of an approved antibiotic.^17^ Here, we report the postapproval, real-world use, and clinical outcomes of cefiderocol therapy in the Veterans’ Health Administration (VHA) from 2019 to 2022 to inform cefiderocol practices for stewardship teams.

## Methods

We conducted a prospective, observational study of patients who received cefiderocol within the VHA. The VHA is the largest integrated healthcare system in the United States and serves >9 million enrolled veterans through its 171 Veterans’ Affairs Medical Centers and community partners.

We applied the following inclusion criteria: patients who received cefiderocol between November 1, 2019, and October 31, 2022, were admitted to an acute-care or long-term care VHA medical facility and received ≥2 days of therapy. Any cefiderocol dosage and frequency was permitted in this study. Only the first eligible episode was included in the outcome analysis.

All-cause mortality was defined as death from any cause. Clinical failure was defined as a composite outcome based on death or presence of signs and symptoms of infection as described previously.^18^ For lower respiratory tract or urinary tract infections, clinical failure was defined as either nonresolution or lack of substantial improvement of baseline signs or symptoms at 30 days from start of therapy or 7 days after end of cefiderocol therapy, whichever was longer.^2,18^ For endovascular infections, clinical failure was defined as ongoing signs and symptoms of infection, or premature discontinuation of study medication, or unplanned use of an alternative antibiotic within 6 months from start of therapy.^19^ For osteomyelitis, clinical failure was defined as ongoing signs and symptoms of infection, any unplanned surgery for the infection within 6 months from start of therapy, premature discontinuation of study medication, or unplanned use of an alternative antibiotic within 6 months from start of therapy. Patients whose antibiotic treatment was terminated due to change in goals of care were classified as clinical failures. Patients who received empiric cefiderocol therapy in the absence of culture data were included, similar to previously published literature.^9^ Microbiological failure was defined as culturing the same organism, as defined by the CDC NHSN,^20^ at least 7 days after start of cefiderocol.

The data source was the VHA Corporate Data Warehouse (CDW) within the VHA’s Veterans’ Informatics and Computing Infrastructure framework. Eligible subjects were identified through drug administration records and through Observational Medical Outcomes Partnership (OMOP) drug era domain. Structured data (eg, *International Classification of Disease, Ninth Revision* and *Tenth Revision*, ICD-9/-10 codes) were extracted from the CDW and were verified through manual chart review. Unstructured data (eg, notes written by the healthcare providers) were extracted through manual review of medical records. Clinical outcomes were assigned by one investigator (E.A.) using chart review. Also, 20% of patient clinical outcomes were audited by a second investigator (A.C.) blinded to the initial assignment; no inconsistencies were identified. Components of the Charlson comorbidity index in the year prior to the drug administration were extracted from CDW data using ICD-9 and ICD-10 codes, and the resulting index was further adjusted using the patient’s age at that time.

Descriptive statistical analyses were reported where nonparametric data were reported as median values with interquartile ranges, and categorical data were reported as value counts and percentage of total observations. Data were analyzed using StataMP version 17 software (StataCorp, College Station, TX).

The Institutional Review Board of Baylor College of Medicine (Houston, TX) and the Michael E. DeBakey VA Medical Center approved this study. A waiver of informed consent was granted for this study.

## Results

**Figure 1.**
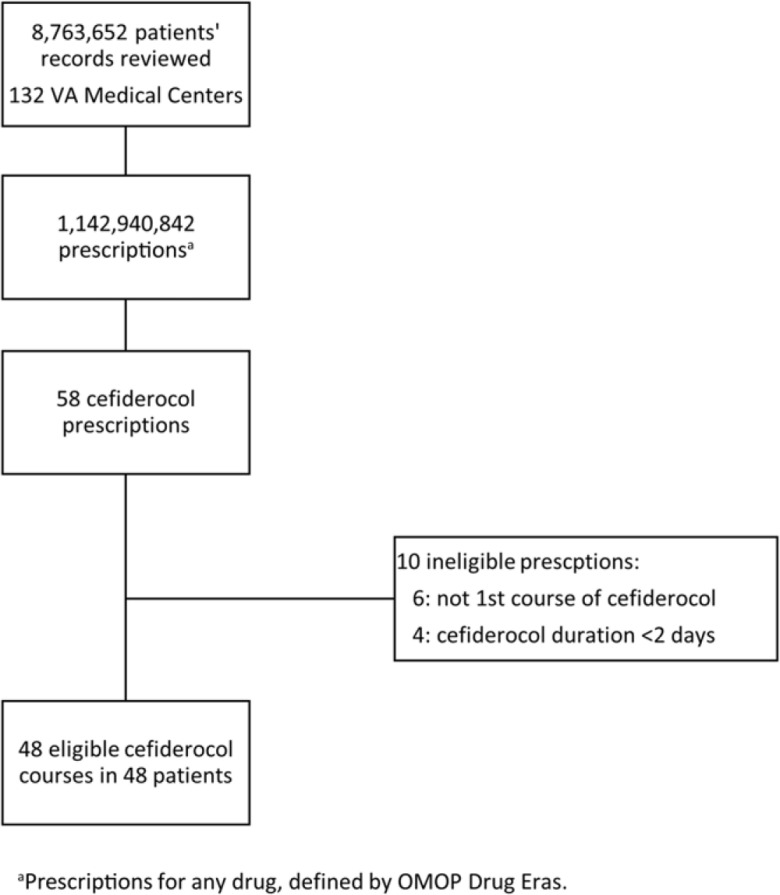
Eligibility assessment.

During the study period, 8,763,652 patients received 1,142,940,842 prescriptions (all drugs, not limited to antibiotics). Among them, 52 patients received 58 courses of cefiderocol (Fig. 1). Moreover, 48 courses in 48 patients met study eligibility. The median age of this cohort was 70.5 years (range, 22–95), 95.8% were male, and the median Charlson comorbidity index was 6 (interquartile range [IQR], 3–9) (Table 1). These statistics were comparable to VHA population with infections from multidrug-resistant organisms in a prior study.^21^ At the onset of infection, 29 (60.4%) of 48 patients were in the intensive care unit, 10 (20.8%) of 48 patients were receiving renal replacement therapy, and 10 (20.8%) had creatinine clearance ≥120 mL/minute (Supplementary Figs. 1 and 2). The most common infectious syndromes were lower respiratory tract infection in 23 (47.9%) of 48 patients and urinary tract infection in 14 (29.2%) of 48 patients. Cefiderocol prescriptions of these 48 patients were initiated median 5 days (IQR, 2–8) after onset of signs or symptoms of infection. Prior to cefiderocol initiation, these patients were most commonly treated with β-lactams and aminoglycosides (see Supplementary Table 3 for details of antimicrobial agents prior to cefiderocol). Prior to cefiderocol therapy, 30 (62.5%) of 48 had never received any novel β-lactam/β-lactamase inhibitor (ie, ceftolozane-tazobactam, ceftazidime-avibactam, meropenem-vaborbactam, or imipenem-cilastatin-relebactam) (Supplementary Tables 1 and 2).

**Table 1.** Clinical Characteristics and Outcomes of Patients Who Received Cefiderocol for Gram-Negative Infections

Characteristics and Outcomes	Total (N = 48), No. (%)^a^
Age, median y (IQR)	70.5 (60.5–74)
Sex, male	46 (95.8)
Charlson comorbidity index, age unadjusted, median (IQR)	6 (3–9)
Charlson comorbidity index, age adjusted, median (IQR)	8.5 (6–11)
Myocardial infarction	12 (25.0)
Congestive heart failure	21 (43.8)
Peripheral vascular disease	22 (45.8)
Cerebral vascular accident or transient ischemic attack	15 (31.3)
Chronic obstructive pulmonary disease	19 (39.6)
Chronic kidney disease, moderate to severe	21 (43.8)
**Diabetes mellitus (all)**	30 (62.5)
Diabetes mellitus, uncomplicated	7 (14.6)
Diabetes mellitus, complicated	23 (47.9)
**Liver disease (all)**	7 (14.6)
Liver disease, mild	5 (10.4)
Liver disease, moderate to severe	2 (4.2)
Peptic ulcer disease	4 (8.3)
Dementia	5 (10.4)
Hemiplegia	11 (22.9)
Connective tissue disease	3 (6.3)
Any malignancy	20 (41.7)
Solid tumor, metastatic	4 (8.3)
AIDS	0 (0.0)
ICU when cefiderocol initiated	29 (60.4)
Renal replacement therapy when cefiderocol initiated	10 (20.8)
Previously received novel BL/BLI, ever^b^	30 (62.5)
Creatinine clearance ≥120 mL/min^c^	10 (20.8)
Days from infection onset to cefiderocol initiation, median (IQR)^d^	5 (2–8)
Days from culture collection to culture finalization, median (IQR)	10 (6–19)
**Cefiderocol**	
Monotherapy therapy	31 (64.6)
Combination therapy	17 (35.4)
Cefiderocol duration of therapy, median (IQR)	8.5 (5–16)
Cefiderocol dose (total g/24 h), median (IQR)	4.5 (2–6)^e^
**Infectious syndrome(s)^f^**	
Lower respiratory tract infection	23 (47.9)
Urinary tract infection	14 (29.2)
Endovascular infection	9 (18.8)
Bone/joint infection	4 (8.3)
Other	8 (16.7)
**Infectious organism(s)** ^**f**^	
*Pseudomonas aeruginosa*	30 (62.5)
Enterobacterales	17 (35.4)
*Acinetobacter baumannii*	10 (20.8)
*Stenotrophomonas maltophilia*	3 (6.3)
Other	2 (4.2)
Negative cultures	3 (6.3)

Note. IQR, interquartile range.

a Data are no. (%) unless otherwise specified.

b Novel β-lactam/β-lactamase inhibitors (BL/BLI) include ceftolozane-tazobactam, ceftazidime-avibactam, meropenem-vaborbactam, imipenem-cilastatin-relebactam.

c FDA package insert recommends increased dosing frequency (2 g every 6 h) compared with standard dosing (2 g every 8 h).

d Time from onset of first infection to first administration of cefiderocol. For patients with multiple infectious syndromes, the earliest infection was analyzed. Three patients initiated cefiderocol at non-VA hospitals and external records documenting onset of infection were not available for review; therefore, these patients were excluded from this analysis.

e Cefiderocol 4.5 g/day corresponds to 1.5 g per 8 h; cefiderocol 2 g/day corresponds to 1 g per 12 h; cefiderocol 6 g/day corresponds to 2 g per 8 h.

f A patient may have one or more infectious syndromes and/or 1 or more organisms. See Supplementary Table for detailed antibiotic susceptibility test results.

The most common pathogens cultured were *P. aeruginosa*, identified in 30 (62.5%) of 48 patients; Enterobacterales, identified in 17 (35.4%) of 48 patients; and *A. baumannii*, identified in 10 (20.8%) of 48 patients. Moreover, 20 patient cultures (41.7%) grew >1 gram-negative isolate, such as gram-negative organisms of a different genus or species or multiple strains of the same organism with different susceptibilities. In total, 77 gram-negative isolates included susceptibility results (Table 2). Carbapenemases were detected in 10 (47.6%) of 21 isolates tested, including 6 isolates harboring *bla*_KPC_, 2 isolates harboring *bla*_NDM_, 1 isolate harboring *bla*_VIM_, and 1 isolate harboring both *bla*_KPC_ and *bla*_NDM_. The initial organism was susceptible to cefiderocol in 20 (83.3%) of 24 isolates tested: 1 *A. baumannii* isolate was resistant, 2 *P. aeruginosa* isolates were intermediately resistant, and 1 isolate *bla*_NDM_-harboring *K. pneumoniae* isolate was intermediately resistant to cefiderocol. The organism was susceptible to carbapenems in 16 (23.2%) of 69 isolates tested, susceptible to ceftolozane-tazobactam in 14 (48.3%) of 29 isolates tested, susceptible to ceftazidime-avibactam in 11 (40.7%) of 27 isolates tested, and susceptible to imipenem-relebactam in 1 (11.1%) of 9 isolates tested. In all patients, cefiderocol treatment was initiated prior to final culture reports (Supplementary Table 4). Notably for these initial cultures, the median time from specimen collection to final culture reports was 10 days (IQR, 6–19). Cefiderocol was started median of 4 days (IQR, 2–7) after specimen collection. Furthermore, 11 (45.8%) of 24 initial cultures with cefiderocol interpretations reported numeric minimum inhibitory concentration (MIC) or zone diameters, and only 1 reported the interpretive criteria standard [eg, Clinical and Laboratory Standards Institute (CLSI), breakpoints, US Food and Drug Administration (FDA) breakpoints, European Committee on Antimicrobial Susceptibility Testing (EUCAST) breakpoints]. Of the 11 cultures with numeric MIC or zone diameters, 4 (44.4%) had MIC or zone diameter values that were interpreted into different categories depending on which interpretative criteria standard was used (Supplementary Table 5). Of these 4 patients, 1 patient was transitioned at day 2 to comfort measures only and died; the other 3 patients were discharged alive.

**Table 2.** Antimicrobial Susceptibility Testing and Molecular Carbapenemase Resistance Testing Results for Gram-Negative Organisms

Agent(s)	*Pseudomonas aeruginosa* (n = 39) Rate, % (Frequency)	Enterobacterales (n = 22) Rate, % (Frequency)^a^	*Acinetobacter baumannii* (n = 10) Rate, % (Frequency)	*Stenotrophomonas maltophilia* (n = 3) Rate, % (Frequency)	Other (n = 2) Rate, % (Frequency)	Total Rate, % (Frequency)
Cefiderocol	**88.2 (15 of 17)**	66.7 (2 of 3)	**85.7 (6 of 7)**	0	0	**85.2 (23 of 27)**
Ceftolozane-tazobactam	45.8 (11 of 24)	33.3 (1 of 3)	0	0	0	44.4 (12 of 27)
Imipenem-relebactam	16.7 (1 of 6)	0 (0 of 1)	0	0	0	14.3 (1 of 7)
Ceftazidime-avibactam	25.0 (4 of 16)	60.0 (6 of 10)	0	0	0	38.5 (10 of 26)
Polymyxins	14.3 (1 of 7)	50.0 (2 of 4)	50.0 (1 of 2)	0	0	30.8 (4 of 13)
Carbapenems	13.5 (5 of 37)	47.4 (9 of 19)	0 (0 of 10)	0	**100 (1 of 1)**	22.4 (15 of 67)
Cefepime	14.7 (5 of 34)	27.8 (5 of 18)	0 (0 of 9)	0	0 (0 of 1)	16.1 (10 of 62)
Ceftazidime	31.0 (9 of 29)	57.1 (4 of 7)	0 (0 of 8)	33.3 (1 of 3)	0 (0 of 1)	29.2 (14 of 48)
Piperacillin-tazobactam	37.5 (12 of 32)	27.8 (5 of 18)	0 (0 of 5)	0	**100 (1 of 1)**	32.1 (18 of 56)
Aminoglycosides	**81.6 (31 of 38)**	70.0% (14 of 20)	50.0 (5 of 10)	0	0 (0 of 1)	72.5 (50 of 69)
Fluoroquinolones	17.9 (7 of 39)	27.3% (6 of 22)	0 (0 of 10)	33.3 (1 of 3)	0 (0 of 1)	18.7 (14 of 75)
Molecular tests	VIM, 1 ND, 7	KPC, 6 KPC & NDM, 1 NDM, 2 ND, 1	ND, 3	None tested	None tested	KPC, 6 NDM, 2 VIM, 1 KPC & NDM, 1 ND, 11

Note. ND, carbapenemase genes not detected by molecular testing. Patients may have 1 or more strains of an organism. For example, a patient with 2 different strains of *P. aeruginosa*, each strain will be included above. Bold denotes sensitivity >80%.

a Includes 6 *K. pneumoniae*, 5 *E. cloacae* complex, 3 *P. mirabilis*, 2 *C. freundii*, *2 E. coli*, 2 *P. stuartii*, 1 *C. farmeri*, 1 *K. oxytoca*.

**Figure 2.**
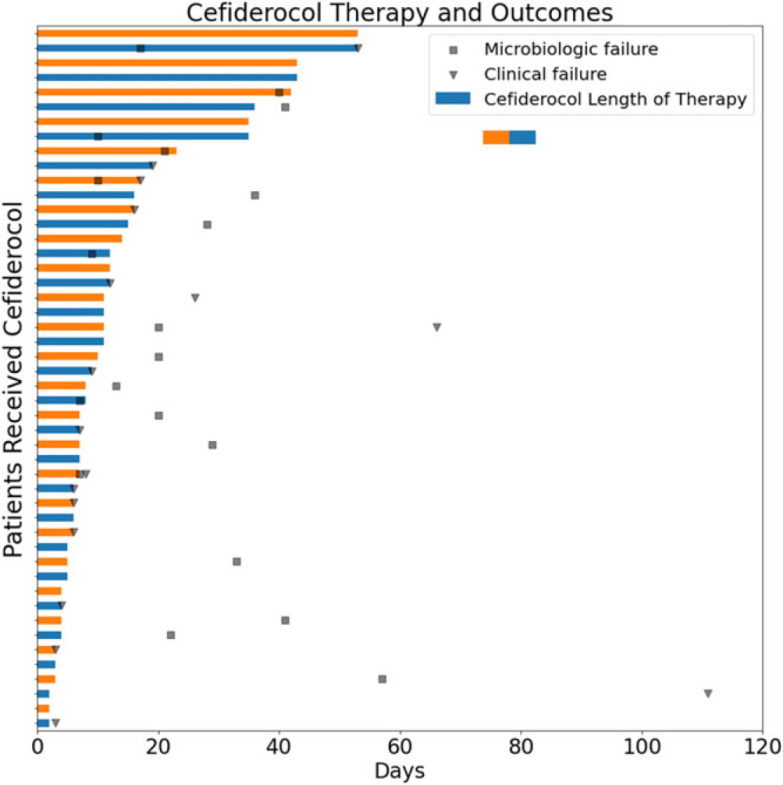
Cefiderocol Therapy and Outcomes.

The median duration of cefiderocol therapy was 8 days (range, 2–53) (Fig. 2), the median cefiderocol dose was 4.5 g per 24 hours (corresponding to 1.5 g per 8 hours), and cefiderocol was used as monotherapy in 31 (64.6%) of these 48 patients (Table 1). The clinical failure rate was 35.4% (17 of 48), and 88.2% (15 of 17) died within 3 days of clinical failure. The 30-day all-cause mortality rate was 27.1% (13 of 48), and the 90-day all-cause mortality rate was 45.8% (22 of 48). Also,12 patients who died had transitioned to comfort or hospice care during treatment and ended the cefiderocol course, with a median of 6 days after starting cefiderocol. The 30-day microbiologic failure rate was 29.2% (14 of 48), and the 90-day microbiologic failure rate was 41.7% (20 of 48) (Tables 3 and 4). Clinical failure rates were high, particularly for endovascular infections (42.9%, 3 of 7) and complicated intra-abdominal infections (100%, 1 of 1) (Table 3). Clinical failure occurred in 30% or higher of cases of urinary tract infections, lower respiratory tract infections, osteomyelitis, and in multisite infections. Mortality accounted for 76.5% (13 of 17) of clinical failures, with 30-day mortality ranging from 18.8% (lower respiratory tract infections) to 100% (complicated intra-abdominal infections). None of the 3 patients with osteomyelitis died within 90 days. The clinical and microbiologic outcomes of patients with polymicrobial infections were similar to patients with monomicrobial infections due to *P. aeruginosa*, Enterobacterales, or *A. baumannii* (Table 4). Among isolates associated with microbiologic failure, 4 patients had 5 isolates that were tested for cefiderocol susceptibility and of these 5 isolates, none had developed resistance to cefiderocol. Only 1 of these cultures reported cefiderocol MIC; the culture associated with microbiologic failure reported cefiderocol MIC of 0.125 µg/mL, and the MIC of the initial culture to cefiderocol was 0.094 µg/mL (Supplementary Table 5).

**Table 3.** Clinical and Microbiologic Outcomes Grouped by Source of Infection

Outcome	Clinical Failure Rate, % (Frequency)	30-Day All-Cause Mortality Rate, % (Frequency)	90-Day All-Cause Mortality Rate, % (Frequency)	30-Day Microbiologic Failure Rate, % (Frequency)	90-Day Microbiologic Failure Rate, % (Frequency)
UTI	30 (3 of 10)	30.0 (3 of 10)	50.0 (5 of 10)	0 (0 of 10)	30.0 (3 of 10)
LRTI	31.3 (5 of 16)	18.8 (3 of 16)	50.0 (8 of 16)	43.8 (7 of 16)	56.3 (9 of 16)
Endovascular infection	42.9 (3 of 7)	28.6 (2 of 7)	42.9 (3 of 7)	42.9 (3 of 7)	42.9 (3 of 7)
Osteomyelitis	33.3 (1 of 3)	0 (0 of 3)	0 (0 of 3)	0 (0 of 3)	0 (0 of 3)
cIAI	100 (1 of 1)	100 (1 of 1)	100 (1 of 1)	0 (0 of 1)	0 (0 of 1)
Other^a^	50.0 (1 of 2)	50.0 (1 of 2)	50.0 (1 of 2)	0 (0 of 2)	0 (0 of 2)
≥2 infection sites	33.3 (3 of 9)	33.3 (3 of 9)	44.4 (4 of 9)	44.4 (4 of 9)	55.5 (5 of 9)
Total	35.4 (17 of 48)	27.1 (13 of 48)	45.8 (22 of 48)	29.2 (14 of 48)	41.7 (20 of 48)

Note. cIAI, complicated intra-abdominal infection; LRTI, lower respiratory tract infection; UTI, urinary tract infection.

a Includes febrile neutropenia, skin-soft tissue infection.

**Table 4.** Clinical and Microbiologic Outcomes Grouped by Organism

Organism	Clinical Failure Rate, % (Frequency)	30-Day All-Cause Mortality Rate, % (Frequency)	90-Day All-Cause Mortality, % (Frequency)	30-Day Microbiologic Failure, % (Frequency)	90-Day Microbiologic Failure, % (Frequency)
*Pseudomonas aeruginosa*	33.3 (6 of 18)	22.2 (4 of 18)	44.4 (8 of 18)	33.3 (6 of 18)	44.4 (8 of 18)
Enterobacterales	37.5 (3 of 8)	37.5 (3 of 8)	50.0 (4 of 8)	0 (0 of 8)	25.0 (2 of 8)
*Acinetobacter baumannii*	50.0 (1 of 2)	50.0 (1 of 2)	50.0 (1 of 2)	50.0 (1 of 2)	50.0 (1 of 2)
*Stenotrophomonas maltophilia*	0 (0 of 2)	0 (0 of 2)	0 (0 of 2)	50.0 (1 of 2)	50.0 (1 of 2)
Other^a^	100 (1 of 1)	100 (1 of 1)	100 (1 of 1)	0 (0 of 1)	0 (0 of 1)
≥2 gram-negative organism^b^	35.7 (5 of 14)	28.6 (4 of 14)	50.0 (7 of 14)	42.9 (6 of 14)	57.1 (8 of 14)
Organism unknown	33.3 (1 of 3)	33.3 (1 of 3)	33.3 (1 of 3)	0 (0 of 3)	0 (0 of 3)
Total	35.4 (17 of 48)	27.1 (13 of 48)	45.8 (22 of 48)	29.2 (14 of 48)	41.7 (20 of 48)

a Gram-negative rod grew in blood culture but failed to grow for species identification.

b Coinfections included *Achromobacter xylosoxidans*, *Acinetobacter baumannii*, Enterobacterales, *Pseudomonas aeruginosa*, Enterobacterales, *Stenotrophomonas maltophilia*.

## Discussion

We have reported the clinical characteristics and outcomes from a nationwide VHA cohort who received cefiderocol to expand the existing published literature on cefiderocol therapy, particularly for treatment of *P. aeruginosa* infections. The key findings of this study include a clinical failure rate of 35.4% (18 of 48) and a 30-day microbiological failure rate of 29.2% (14 of 48). Of the 13 patients who died within 30 days of initiation of cefiderocol, 11 had transitioned to hospice or comfort care, therefore stopping antibiotics. This situation reflects the real-world nature of the use of cefiderocol and the severity of illness and comorbidity burden among patients receiving this medication. All-cause mortality, although precise, cannot elucidate whether the antibiotic given was ineffective or whether the overall patient condition or complications of the infection were the determining factors leading to death.

Of 48 patients prescribed cefiderocol in this study, 20 (41.6%) were at the extremes of renal function, including creatinine clearance ≥120 mL/minute or receiving renal replacement therapy. Therefore, they required increased administration frequency compared with standard dosing (ie, 2 g every 6 hours rather than 2 g every 8 hours) or dose adjustments based on effluent flow rate of continuous renal replacement therapy. Notably, augmented renal clearance (creatinine clearance >120 mL/minute) can be associate with critical illness but can also occur in non–critically ill patients.^22^ This study also found high rates of cefiderocol susceptibility among initial causative pathogens, when tested (83.3%, 20 of 24). Overall, patients who received cefiderocol were medically complex and often required management by intensivists, infectious disease specialists, medical microbiologists, and clinical pharmacy specialists.

The study has several strengths that expand the literature on the effectiveness of cefiderocol. First, the larger cohort size compared with the existing published literature.^6–13^ Second, this study captured real-world prescribing of cefiderocol after US FDA approval rather than clinical trials or compassionate use programs. Third, this study measured outcomes of a cohort of patient with *P. aeruginosa* as the most common pathogen, whereas the existing literature largely reports on *A. baumannii* infections. After the randomized controlled trials examining cefiderocol^2–4^ were performed, only small studies have evaluated the effectiveness of cefiderocol. Only 2 small studies reported results in which *P. aeruginosa* was the most common pathogen.^12,13^ Meschiari et al^12^ examined 17 patients with *P. aeruginosa* infection treated with cefiderocol through a compassionate-use protocol and reported a 30-day all-cause mortality rate of 23.5% and a 90-day all-cause mortality rate of 35.3%. Both rates are in line with the results of this study. Bleibtreu et al^13^ reported outcomes of 13 patients, 10 of whom had infections with *P. aeruginosa*, treated with cefiderocol through a compassionate-use protocol and reported a 23.1% mortality rate.^13^

Another 5 studies have been conducted under real-world prescribing conditions.^7,9,10,14,15^ Falcone et al^14,15^ reported a 28-day all-cause mortality rate of 22.2% (4 of 18) associated with cefiderocol therapy of metallo-β-lactamase–producing Enterobacterales. In a second study, the 30-day mortality rate was 34% (16 of 47) in patients with *A. baumannii* infections treated with cefiderocol. In a study by Gavaghan et al,^10^ the 30-day mortality rate was 42% (10 of 24) in real-world cefiderocol therapy for patients among whom pneumonia was the most common source of infection. *A. baumannii* was the most common organism in the Advocate Aurora Health system, which comprises hospitals in Wisconsin and Illinois.^9^ In a single center in France, Hoellinger et al^10^ reported a 30-day mortality rate of 60% in 10 patients, most of whom were highly immunosuppressed (eg, organ transplantation, acute hematologic malignancy, metastatic solid tumor), treated with cefiderocol for nonfermenting gram-negative bacilli infections, of which 6 of 10 were due to *P. aeruginosa*. In another single-center study in France, Rando et al^7^ reported a 46.1% mortality rate among 13 patients with *A. baumannii* pneumonia, of whom all but one also had COVID-19,.^7^ Also, 4 other studies reported outcomes with cefiderocol therapy for *A. baumannii* infections; they were conducted under compassionate use programs in Italy with mortality rates of 10% (1 of 10), 23.1% (3 of 13), 55% (23 of 42), and 30.8% (4 of 13).^6,8,11,16^

Our study had several limitations. First, this was a descriptive study, and we did not compare cefiderocol to alternative therapies. Therefore, we could not determine whether cefiderocol was associated with increased mortality compared to other available therapies. This question likely could only be adequately addressed by a randomized clinical trial even larger than the CREDIBLE-CR phase 3 clinical trial.^2^ Previously published studies used different inclusion criteria and outcomes definitions, and we sought to use the most widely recognized criteria and definitions (eg, assessing mortality), but for those without consensus in the literature, we selected definitions based on clinical relevance. For example, the duration of cefiderocol therapy for inclusion in prior studies ranged from a single dose^13^ to at least 5 days^9^; we chose 2 days similar to Hoellinger et al^10^ and Falcone et al.^14,15^ When restricting our cohort only to patients who received ≥5 days cefiderocol, the rates of 30-day all-cause mortality (26.3%; 10 of 38) and 90-day all-cause mortality (44.7%; 17 of 38) were not different from the overall cohort. Second, antimicrobial susceptibility testing was not standardized, and multidrug-resistant isolates each underwent susceptibility testing with different panels of antibiotic. Also, interpretive breakpoint criteria were at the discretion of the reporting laboratory. For example, the cefiderocol susceptible breakpoint for *P. aeruginosa* for the US FDA, EUCAST, and CLSI are ≤1 µg/mL, ≤2 µg/mL and ≤4 µg/mL, respectively.^23–25^ Of the cultures that reported numeric MIC or zone diameters, we found high rates (44.4%; 4 of 11) of isolates where the interpretation (S/I/R) would differ based on which breakpoints (ie, CLSI, US FDA, or EUCAST) were applied. This high rate warrants further monitoring and investigation to determine whether some form of bias (eg, reporting bias) was present; for now, we believe antimicrobial stewardship teams and medical microbiologists should consider reporting the numeric cefiderocol MIC or zone diameters, the breakpoints applied, and carefully consider how to manage isolates with MIC or zone diameters where the agencies (CLSI, US FDA, and EUCAST) have differing interpretations. This investigation reflects the real-world diversity in microbiology laboratory practices and the complexities of implementing cefiderocol susceptibility testing, particularly soon after FDA approval.^26^ Notably, all subjects had cefiderocol initiated prior to availability of final cefiderocol AST results. There have been reports of cefiderocol-resistant isolates in patients who never received cefiderocol^27^ and isolates collected prior to its approval by the US FDA,^28^ but resistance rates were low in other surveillance.^29^ Third, this study was conducted within the VHA; therefore, patients were within the United States and were predominantly male.

In summary, we have reported the clinical failure rates, microbiological failure rates, and all-cause mortality in a cohort of patients with significant comorbidities who received cefiderocol. All of these failures rates and mortality rates were high, which probably reflects the underlying comorbidities of the patients and the refractory nature of their underlying infections. Patients prescribed cefiderocol frequently had complex pharmacodynamics, which is an opportunity for stewardship teams to ensure correct cefiderocol dosing. We also detected high rates of isolates with cefiderocol MIC or zone diameters, which would have different cefiderocol antimicrobial susceptibility testing interpretations depending on which agency’s breakpoints are used. We recommend that antimicrobial stewardship teams and medical microbiologists consider routinely reporting cefiderocol MIC or zone diameters and which agency’s breakpoints are used. Cefiderocol provides a new therapeutic option for patients with multidrug-resistant gram-negative infections and should be further investigated.

## Supplementary material

For supplementary material accompanying this paper visit http://doi.org/10.1017/ash.2023.165. click here to view supplementary material
