# Supplementary material for: Real-world experience with cefiderocol therapy for Pseudomonas aeruginosa and other multidrug resistant gram-negative infections within the Veterans Health Administration, 2019–2022
Source: Antimicrob Steward Healthc Epidemiol. 2023 May 4;3(1):e90. doi: 10.1017/ash.2023.165 (PMC10173288; doi:10.1017/ash.2023.165)
Supplement: Supplementary file 1 [file S2732494X23001651sup001.docx]

**Supplemental Figure 1: Renal function at start of cefiderocol therapy, estimated by eGFR and CLcr**

Patients receiving renal replacement therapy were assigned eGFR and CLcr values of 1.

Qualitative data were converted to the closest integer (example: an eGFR value extracted as “>60” was transformed to 61 mL/min/1.73m^2^ for this figure.

CLcr: creatinine clearance by Cockcroft-Gault.

eGFR: estimated glomerular filtration rate (mL/min/1.73m^2^) by MDRD or CKD-EPI, as reported in the database.

**Supplemental Figure 2: Treatment location at cefiderocol initiation**

ICU: intensive care unit

MICU: medical intensive care unit

SICU: surgical intensive care unit

SCI: spinal cord injury unit

Med/Surg: acute care ward, non-ICU, providing care for medical and/or surgical patients

**Supplemental Table 1: Most recent administration of novel BL/BLI prior to cefiderocol therapy**

| Timeframe | Ceftazidime/  avibactam | Ceftolozane/  tazobactam | Meropenem/  vaborbactam | Imipenem/  relebactam |
| --- | --- | --- | --- | --- |
| <10 days prior to cefiderocol therapy | 9 | 4 | 0 | 1 |
| >10 days to <30 days prior to cefiderocol therapy | 2 | 0 | 0 | 0 |
| >30 days to <1 year prior to cefiderocol therapy | 1 | 3 | 0 | 0 |
| >1 year to <2 years prior to cefiderocol therapy | 1 | 1 | 1 | 0 |
| >2 years prior to cefiderocol therapy | 1 | 0 | 0 | 0 |
| Total | 14 | 8 | 1 | 1 |

Seven patient received >2 novel BL/BLI treatments prior to cefiderocol.

Novel beta-lactam/beta-lactamase inhibitors (BL/BLI) include ceftolozane/tazobactam (C/T), ceftazidime/avibactam (C/A), meropenem/vaborbactam (M/V), imipenem/cilastatin/relebactam (M/V).

**Supplemental Table 2: Seven patients who received multiple courses of novel BL/BLI prior to cefiderocol therapy**

|  | <10 days prior to cefiderocol therapy | >10 days to <30 days prior to cefiderocol therapy | >30 days to <1 year prior to cefiderocol therapy | >1 year to <2 years prior to cefiderocol therapy | >2 years prior to cefiderocol therapy |
| --- | --- | --- | --- | --- | --- |
| 1 | None | None | None | C/A | C/A x2 courses |
| 2 | None | None | C/A x2 courses  C/T x3 courses | None | None |
| 3 | None | None | None | None | C/A  M/V |
| 4 | C/A | None | C/A  C/T | None | None |
| 5 | C/A | None | C/T | None | None |
| 6 | I/R | C/A | None | None | None |
| 7 | C/A | None | C/A | C/A  C/T | None |

**Supplemental Table 3: Antimicrobial regimens prior to initiation of cefiderocol**

| Antimicrobial class | N = 47 [1] |
| --- | --- |
| Beta-lactam, any [2]  Ampicillin/sulbactam  Amoxicillin/clavulanate  Aztreonam  Cefazolin  Cefepime  Cefpodoxime  Ceftazidime  Ceftazidime/avibactam  Ceftolozane/tazobactam  Ceftriaxone  Imipenem/relebactam  Meropenem  Piperacillin/tazobactam | 42  6  1  1  1  15  1  3  12  5  5  1  14  9 |
| Aminoglycoside, any [2]  Amikacin  Gentamicin | 9  6  4 |
| Tetracycline, any [2]  Minocycline  Tigecycline | 6  2  4 |
| Fluoroquinolone, any [2]  Ciprofloxacin  Levofloxacin | 8  6  2 |
| Other [2]  Ciprofloxacin otic drops  Colistin  Polymyxin B  Trimethoprim/sulfamethoxazole | 1  1  1  3 |

[1] one patient excluded due to non-VA records without documentation antimicrobials prior to cefiderocol.

[2] A patient may receive more than one antimicrobial class, and may more than one agent within any class.

**Supplemental Table 4: Cefiderocol regimens in combination with other antibiotics**

| Antibiotic co-administered with cefiderocol | Number of subjects |
| --- | --- |
| Aminoglycosides, systemic  Amikacin  Tobramycin | 7  6  1 |
| Ampicillin/sulbactam | 1 |
| Fluoroquinolone  Ciprofloxacin  Levofloxacin | 3  2  1 |
| Tetracyclines  Tigecycline  Minocycline | 5  3  2 |
| Trimethoprim/sulfamethoxazole | 1 |
| Other: ceftazidime/avibactam & aztreonam & azithromycin | 1 |

One patient received regimen of cefiderocol, amikacin, and rifampin. One patient receive cefiderocol, ciprofloxacin, and tobramycin.

**Supplemental Table 5: Cefiderocol Microbiology Report Values and Metadata**

|  | Days from culture specimen collection to cefiderocol initiation | Days after culture collection to culture report final | Days cefiderocol initiation prior to report final | Cefiderocol AST Value | Cefiderocol AST interpretation | Notes |
| --- | --- | --- | --- | --- | --- | --- |
| P1 | 0 | 8 | 8 | n/r | S |  |
| P2 | 0 | 18 | 18 | n/r | I |  |
| P3 | 1 | 8 | 6 | n/r | S |  |
| P4 | 1 | 19 | 17 | 0.06 mcg/mL | S |  |
| P5 | 2 | 3 | 1 | 20 mm | S [1] |  |
| P6 | 2 | 6 | 3 | n/r | S |  |
| P7 | 2 | n/r | n/a | 16 mm | I [2] |  |
| P8.1 | 3 | 6 | 3 | 0.094 mcg/mL | S | Culture from initial infection. |
| P8.2 | n/a | 6 | n/a | 0.094 mcg/mL  0.125 mcg/mL | S | Culture from microbiologic failure. Two colony morphologies tested. |
| P9 | 3 | 8 | 4 | 8 mcg/mL | I [3] |  |
| P10.1 | 4 | 4 | 0 | n/r | S | Culture from initial infection. |
| P10.2 | n/a | 5 | n/a | n/r | S | Culture from microbiologic failure. |
| P10.3 | n/a | 4 | n/a | n/r | S | Culture after microbiologic failure. |
| P11 | 4 | 6 | 2 | 17 mm | S [4] |  |
| P12 | 4 | 7 | 3 | 0.5 mcg/mL | S |  |
| P13 | 4 | 13 | 8 | 0.5 mcg/mL | S |  |
| P14 | 4 | 17 | 13 | n/r | S |  |
| P15 | 5 | 10 | 5 | n/r | S |  |
| P16 | 5 | 40 | 35 | n/r | S |  |
| P17 | 6 | 6 | 9 | 27 mm | S |  |
| P18.1 | 7 | 8 | 0 | n/r | S | Culture from initial infection. |
| P18.2 | n/a | 3 | n/a | n/r | S | Culture from microbiologic failure. |
| P19 | 7 | 13 | 6 | 0.25 mcg/mL | S |  |
| P20 | 7 | 19 | 11 | n/r | S |  |
| P21 | 7 | 25 | 18 | n/r | S |  |
| P22 | 7 | 55 | 47 | >64 mcg/mL | R |  |
| P23 | 8 | 15 | 7 | n/r | S |  |
| P24 | 8 | 35 | 27 | n/r | S |  |
| P25.1 | n/r | n/r | n/r | n/r | n/a | Culture from initial infection. |
| P25.2 | n/a | 6 | n/r | n/r | S | Culture from microbiologic failure. |

S: susceptible; I: intermediate; R: resistant.

n/a: not applicable.

n/r: not reported

[1] Zone diameter of 20 mm against the organism (*P. aeruginosa*) is Susceptible using CLSI breakpoints; FDA breakpoints would interpret as Intermediate; EUCAST breakpoints would interpret as Resistant.

[2] Zone diameter of 16 mm against the organism (*P. aeruginosa*) is Intermediate using CLSI & FDA breakpoints; EUCAST breakpoints would interpret as Resistant.

[3] MIC of 8 mcg/mL against *P. aeruginosa* is Intermediate using CLSI breakpoints; FDA breakpoints would interpret as Resistant; EUCAST breakpoints would interpret as Resistant.

[4] Zone diameter of 17 mm against the organism (*Enterobacter cloacae* complex) is Susceptible using CLSI breakpoints; FDA recognizes CLSI breakpoints for Enterobacterales; EUCAST breakpoints would interpret as Resistant.
